# Squeeze-EnGAN: Memory Efficient and Unsupervised Low-Light Image Enhancement for Intelligent Vehicles

**DOI:** 10.3390/s25061825

**Published:** 2025-03-14

**Authors:** Haegyo In, Juhum Kweon, Changjoo Moon

**Affiliations:** 1 Department of Smart Vehicle Engineering, Konkuk University, Seoul 05029, Republic of Korea; inkyo245@konkuk.ac.kr; 2 Graduate School of Future Defense Technology Convergence, Konkuk University, Seoul 05029, Republic of Korea; jkweon@konkuk.ac.kr

**Keywords:** autonomous driving, low-light image enhancement, unsupervised learning, generative adversarial network

## Abstract

Intelligent vehicles, such as autonomous cars, drones, and robots, rely on sensors to gather environmental information and respond accordingly. RGB cameras are commonly used due to their low cost and high resolution but are limited in low-light conditions. While employing LiDAR or specialized cameras can address this issue, these solutions often incur high costs. Deep learning-based low-light image enhancement (LLIE) methods offer an alternative, but existing models struggle to adapt to road scenes. Furthermore, most LLIE models rely on supervised training but are heavily constrained by the lack of low-light and normal-light paired datasets. In particular, obtaining paired datasets for driving scenes is extremely challenging. To address these issues, this paper proposes Squeeze-EnGAN, a memory-efficient, GAN-based LLIE method capable of unsupervised learning without paired image datasets. Squeeze-EnGAN incorporates a fire module into a U-net architecture, substantially reducing the number of parameters and Multiply-Accumulate Operations (MACs) compared to its base model, EnlightenGAN. Additionally, Squeeze-EnGAN achieves real-time performance on devices like Jetson Xavier (0.061 s). Significantly, enhanced images improve object detection performance over original images, demonstrating the model’s potential to aid high-level vision tasks in intelligent vehicles.

## 1. Introduction

In autonomous driving systems, vehicles acquire information about their surroundings through various sensors. Among these sensors, RGB cameras are highly sought after due to their ability to capture high-resolution information and their relatively low cost. Furthermore, the color of an object is a critical feature for distinguishing objects, and cameras are the only sensors capable of capturing color information. For this reason, RGB cameras are widely utilized in tasks such as object detection, object tracking, semantic segmentation, and visual SLAM (Simultaneous Localization and Mapping) [1,2,3] in autonomous vehicles and intelligent mobility systems. Additionally, active research is being conducted on LiDAR-camera sensor fusion to identify object categories that are challenging to discern using LiDAR alone [4,5].

However, due to the characteristics of camera sensors, their performance is highly sensitive to ambient lighting conditions, which can lead to performance degradation. In particular, RGB cameras struggle to identify objects in low-light environments, such as during nighttime or in areas without light sources. This issue poses significant risks for intelligent mobility systems driving at night, as they may fail to respond appropriately to dynamic objects like pedestrians or other vehicles, as well as static obstacles such as poles, buildings, vegetation, and potholes [6]. Additionally, autonomous vehicles may fail to recognize visual cues such as lane markings or traffic signs, potentially resulting in violations of traffic regulations. While the vehicle’s headlights can serve as a built-in light source to detect objects ahead, their effective range is limited. Furthermore, in low-light conditions, the intensity of received light is diminished, leading to a reduced signal-to-noise ratio and increased susceptibility to noise.

To address this issue, this study developed a deep learning-based low-light image enhancement (LLIE) model to overcome the limitations of RGB cameras in autonomous driving environments. In particular, the main objective was to design lightweight LLIE models suitable for autonomous driving applications. Since reaction speed to surrounding objects is crucial in autonomous driving systems, all stages—sensor input, object recognition, decision-making and path generation, and control—must be executed in rapid cycles [7]. If the computational resources required for the driving process (e.g., computation time and memory) are excessive, the decision-making cycle may slow down, impairing the system’s ability to respond swiftly to sudden obstacles or environmental changes. The entire autonomous driving process typically consists of numerous sub-tasks, and LLIE can be considered one of these sub-tasks. If a single task consumes fewer resources while meeting the real-time computational constraints, other tasks can allocate more resources for complex calculations or incorporate new functionalities. This highlights the importance of reducing computation time for LLIE as a critical challenge in integrating it into the autonomous driving process. This challenge becomes even more significant given that most intelligent mobility systems operate within edge computing environments [8,9].

We identified the following challenges in applying existing low-light image enhancement (LLIE) research to autonomous driving environments:

1. General LLIE models struggle to adequately reflect the characteristics of driving environments. Issues such as noise, overexposure, and blur make it difficult to reliably apply these models to autonomous driving.
2. Most deep learning-based LLIE approaches rely on supervised learning methods that require paired normal and low-light images for training. However, obtaining natural image pairs for the same scene is challenging, particularly for driving scenarios where creating such pairs is even more constrained.
3. For LLIE to be applied to autonomous driving, computational resource usage must be minimized, but few models prioritize this requirement.

To address these issues, this study proposes Squeeze-EnGAN, an LLIE model designed to be reliably applied to autonomous driving systems. The foundation of Squeeze-EnGAN is EnlightenGAN [10], which first demonstrated high-performance LLIE without the need for paired images through the GAN [11] architecture. By inheriting the advantage of training on single images, Squeeze-EnGAN is robust across diverse driving scenarios using data captured in various environments.

Additionally, Squeeze-EnGAN incorporates the fire module proposed in SqueezeNet [12] to make the U-net [13] generator network more lightweight, addressing the computational constraints of edge computing environments. Compared to EnlightenGAN, Squeeze-EnGAN significantly reduces the number of parameters and computational overhead. Experimental results using an on-board computer demonstrate the effectiveness of this lightweight design by comparing inference times. Moreover, a comparative analysis of output image quality confirms that the model achieves minimal performance degradation despite its lightweight design.

## 2. Related Works

### 2.1. Traditional Methods

To overcome the limitations of RGB cameras and obtain high-quality images, LLIE techniques have been continuously studied. Traditional LLIE methods can be broadly categorized into hardware-based and software-based approaches.

Hardware-based methods include providing additional light sources, such as using a flash during capture, or increasing the exposure time to allow the camera to receive more light. However, in autonomous driving environments, adding external light sources is often impractical. Furthermore, increasing the exposure time can result in motion blur, which degrades object recognition performance due to afterimages of moving subjects.

In contrast, software-based methods modify the captured data rather than the imaging process itself. For instance, histogram equalization [14], which redistributes the pixel intensity distribution to achieve a more uniform histogram, is a commonly used technique. Another widely studied approach employs the Retinex theory [15], which postulates that an image can be decomposed into illumination and reflectance components. In this approach, the brightness of the illumination component is enhanced, and noise in the reflectance component is reduced. However, these methods often struggle to deliver consistent performance under varying low-light conditions, limiting their applicability to autonomous driving environments, where the system must adapt to diverse scenarios.

### 2.2. Deep Learning Approaches

Advancements in computing hardware have accelerated progress in the field of artificial intelligence, leading to the application of deep learning techniques in low-light image enhancement. Early studies incorporating deep learning, such as LLNet [16], demonstrated superior performance compared to traditional methods, and since then, a variety of neural network models have been proposed.

Supervised learning methods for low-light image enhancement require training datasets consisting of pairs of low-light images and their corresponding normal-light counterparts captured under identical conditions. This approach has been widely adopted in most neural networks due to its superior performance compared to other methods. However, in real-world scenarios, obtaining perfectly paired low-light and normal-light images of the same scene is highly challenging.

In contrast, unsupervised learning methods rely on independent images rather than paired datasets for training, thereby eliminating the data pairing constraints faced by supervised approaches. EnlightenGAN [10], the first neural network to utilize an unsupervised approach, employs a GAN [11]-based architecture. EnlightenGAN offers the significant advantage of enabling training with unpaired images.

For intelligent mobility systems, which operate in diverse environments, it is essential to apply robust models capable of adapting to various conditions. Therefore, this study adopts an unsupervised approach, leveraging unpaired and diverse environmental training datasets to enhance adaptability and robustness.

### 2.3. Adversarial Learning Without Supervision

The Generative Adversarial Network (GAN) [11], proposed by Ian Goodfellow, is a model that consists of two sub-network structures: a generator and a discriminator. GAN employs a training strategy where the generator and discriminator compete against each other. The generator takes random noise vectors as input and learns to produce outputs that mimic real images. In contrast, the discriminator receives either the generator’s output or a real image as input and learns to distinguish whether the input is a real image. As the two networks continue to compete during training, the generator’s outputs become increasingly similar to real images.

Subsequent developments, such as Deep Convolutional GAN (DCGAN) [17], applied convolutional layers and marked significant progress in tasks like image synthesis and style transfer. GANs have also demonstrated their utility in low-level vision tasks, including super-resolution [18], denoising [19], and dehazing [20]. Some studies [21,22] have proposed unsupervised models that learn domain mapping without the need for paired data. In this context, EnlightenGAN [10] has shown high performance in LLIE tasks by using an unsupervised GAN framework, eliminating the need for paired data.

Furthermore, EnlightenGAN utilizes a one-path GAN structure, which requires less computation compared to models employing cycle-consistency. However, EnlightenGAN was not designed with a focus on model lightweighting. To address this limitation, this study proposes a more resource-efficient and lightweight model to improve computational efficiency.

### 2.4. U-net with Fire Module

**Figure 1:**
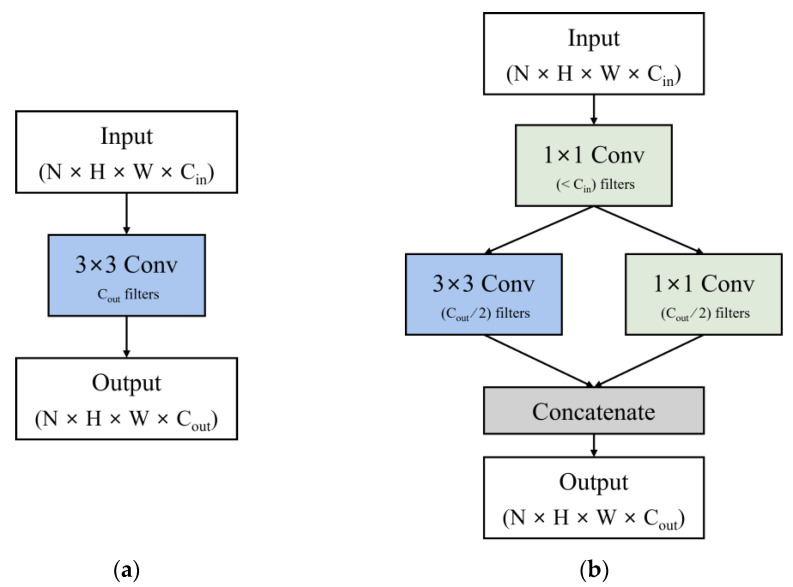
(**a**) represents the input and output of a conventional convolution layer with a kernel size of 3 × 3; (**b**) represents the structure of the fire module, which can replace the 3 × 3 convolution layer.

SqueezeNet [12] proposed the fire module to improve the memory efficiency of convolutional neural networks and reduce real-time inference latency. The fire module is a lightweight alternative to a single convolutional layer and is structured as shown in Figure 1. A standard convolution layer, illustrated in Figure 1a, contains filters equal to the number of specified output channels, with each filter applied across all input channels to produce a corresponding output channel. As a result, the number of channels increases to match the number of output channels. On the other hand, the fire module consists of two layers, as shown in Figure 1b. The first layer is a 1 × 1 convolutional layer that squeezes the input channels to a predefined number of intermediate channels. The number of intermediate channels is usually set to be smaller than the number of input channels. The feature maps produced by the first layer are simultaneously passed to two parallel convolutional layers: one is a 1 × 1 convolutional layer and the other is a 3 × 3 convolutional layer. The number of output channels from each of these two layers is set to half of the final output size and the final output of the fire module is obtained by concatenating the feature maps produced by both layers.

The fire module employs the following strategies to enable more efficient computation compared to conventional convolutional layers: (1) Replacing some 3 × 3 filters with 1 × 1 filters. This reduces the number of parameters to approximately one-ninth of the original. (2) Reducing the number of input channels for the 3 × 3 filters. Since the input channels are squeezed using 1 × 1 filters before being processed by the 3 × 3 filters, fewer channels require computation in the 3 × 3 layer. Using these strategies, SqueezeNet achieved AlexNet-level accuracy with a model size of less than 0.5 MB.

Squeeze U-net [23] is a study that applied the fire module to the U-net [13] architecture to create a lightweight version of U-net. This approach retained the original model’s semantic segmentation performance while reducing memory usage and computation time. Inspired by this approach, the present study replaces the U-net generator network in EnlightenGAN with a Squeeze U-net structure, developing a lightweight model that maintains performance and is more suitable for onboard systems.

## 3. Methods

Inspired by EnlightenGAN [10], the overall architecture of Squeeze-EnGAN comprises a generator with a U-net [13] structure and a global-local discriminator. This design enables training with unpaired images and allows the model to adapt to datasets from diverse environments. Furthermore, Squeeze-EnGAN incorporates the Squeeze U-net [23] approach, which utilizes fire modules in the generator network. This integration facilitates memory-efficient LLIE tasks.

### 3.1. Generator with Fire Modules

**Figure 2:**
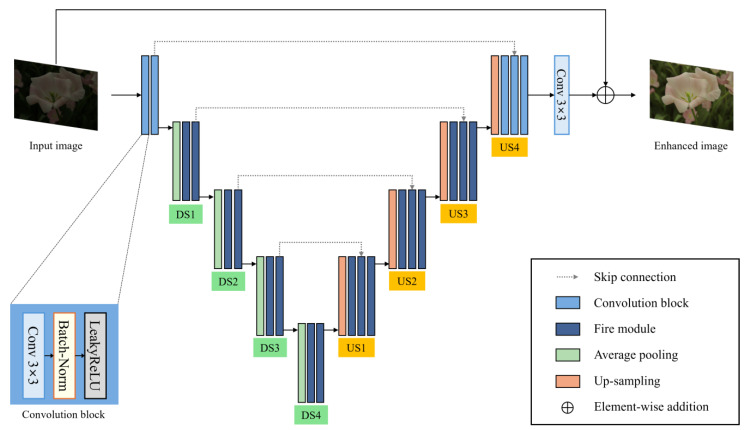
The generator network of Squeeze-EnGAN. The contracting path consists of four downsampling units (DS), while the expansive path comprises four upsampling units (US). The features extracted in the contracting path are concatenated with the corresponding layers in the expansive path via skip connections.

The generator is based on an encoder-decoder structure derived from U-net [13] and the overall structure is illustrated in Figure 2. U-net is a model that has achieved significant success in the field of image semantic segmentation. It consists of an encoder that extracts features from the input image and a decoder that reconstructs a semantic representation at the original resolution from the compressed features. U-net is effective due to its use of skip connections, which help preserve high-resolution information by directly connecting corresponding encoder and decoder layers. This innovative idea has led to the development of many derivative models and has also been applied in the fields of image generation and restoration. The generator of Squeeze-EnGAN also extracts features from the original low-light image and reconstructs it into a normal-light image. As shown in Figure 2, the architecture consists of an encoder that performs down-sampling and a decoder that performs up-sampling, with skip connections linking corresponding layers between the encoder and decoder.

However, the generator of Squeeze-EnGAN replaces the standard convolution layers with fire modules [12] throughout the encoder and decoder, except for the initial layers of the encoder and the final layers of the decoder. Each fire module consists of a 1 × 1 convolution layer followed by parallel 3 × 3 and 1 × 1 convolution layers, as shown in Figure 1. The initial 1 × 1 convolution layer compresses the input channels and passes them to the subsequent convolution layers. The feature maps produced by the 3 × 3 and 1 × 1 convolution layers are concatenated to form the output of the fire module.

In the encoder, average pooling is used to progressively reduce the size of the input feature maps. During the decoder’s upsampling process, the transpose convolution layers are replaced with an upsample operation followed by a fire module to avoid checkerboard artifacts [24]. The detailed layer specifications of the generator network are provided in Appendix A.

The generator in EnlightenGAN [10] uses a self-regularized attention map derived by inverting the illumination channel of the input image to focus more on locally dark regions. However, this approach requires additional computations for the attention module and incurs overhead during preprocessing, where the illumination map is generated for each input. Squeeze-EnGAN eliminates the use of the attention module, thereby saving the computational resources consumed in this process.

As shown in Table 1, we compared the Multiply-Accumulate Operations (MACs) and the number of parameters for each layer of the generators in EnlightenGAN and Squeeze-EnGAN. The generator with fire modules achieved a significant reduction in computational complexity compared to the original model, with the total MACs reduced by a factor of 0.39 and the number of parameters reduced by a factor of 0.15. This demonstrates that our model achieves a significantly more lightweight design compared to the previous model.

### 3.2. Global-Local Discriminator

The discriminators take real images (real-world images) and fake images (the output images from the generator) as input. The discriminator network is trained to output a value close to 1.0 for real images and a value close to 0.0 for fake images. The discriminator’s output is used not only to update the discriminator itself by minimizing classification error but also to guide the training of the generator through adversarial loss, encouraging the generator to produce images that are increasingly indistinguishable from real ones.

**Figure 3:**
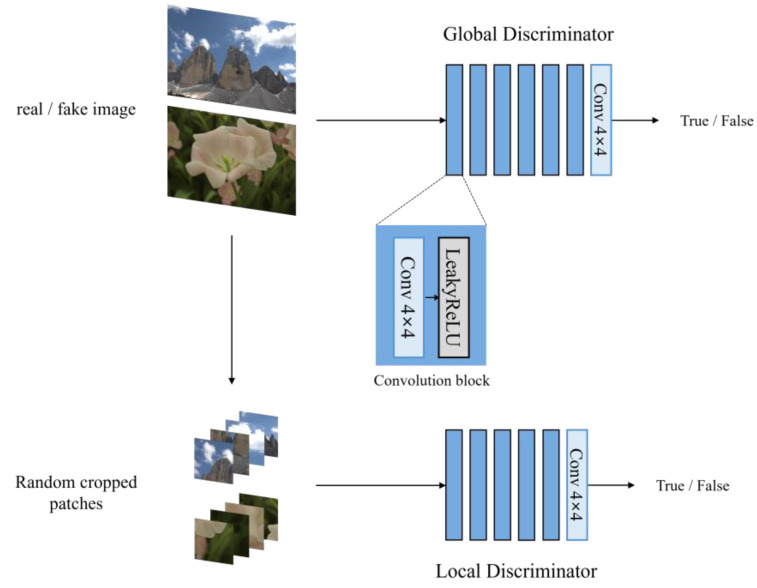
The structure of the global and local discriminator networks. The local discriminator uses randomly cropped regions from the input image of the global discriminator as its input.

Conventional GAN models use the discriminator’s output to minimize the disparity between the distributions of the generator’s output and real images. In contrast, GAN models applied to LLIE aim to reduce the distribution gap between the generator’s output and normal-light images. Furthermore, as demonstrated in previous research [10], a local discriminator is additionally employed to mitigate the impact of locally exaggerated illumination (e.g., small light sources in a dark background). Therefore, we employ two discriminators to enhance the performance of the generator, as illustrated in Figure 3.

The global discriminator takes a full-resolution image as input and processes it through a series of convolutional layers with progressively increasing receptive fields. The local discriminator, on the other hand, uses randomly cropped regions from the global discriminator’s input images, enabling the generator to enhance local quality. Notably, object detection research [25,26] has leveraged local features to detect small objects effectively. Similarly, the local discriminator reinforces detailed image contexts, aiding the detection of objects located in local regions during high-level vision tasks in autonomous driving.

The adversarial loss computed from the output of the global discriminator trains both the generator and the global discriminator. In this process, the loss combines the Relativistic Discriminator [27] and Least Square GAN (LSGAN) [28] methods. The equations for the loss functions applied to the global discriminator *D* and the generator *G* are as follows:

(1) $$L_{D}^{Global}=E_{x_{r}\sim P_{real}}[{\left(D(x_{r})-E_{x_{f}\sim P_{fake}}[D(x_{f})]-1\right)}^{2}]+E_{x_{f}\sim P_{fake}}[{\left(D(x_{f})-E_{x_{r}\sim P_{real}}[D(x_{r})]\right)}^{2}]$$

(2) $$L_{G}^{Global}=E_{x_{f}\sim P_{fake}}[{\left(D(x_{f})-E_{x_{r}\sim P_{real}}[D(x_{r})]-1\right)}^{2}]+E_{x_{r}\sim P_{real}}[{\left(D(x_{r})-E_{x_{f}\sim P_{fake}}[D(x_{f})]\right)}^{2}]$$

In Equations (1) and (2), *D* represents the network of the global discriminator, while $x_{r}$ and $x_{f}$ denote samples of real images (natural normal-light images) and fake images (the output images from the generator), respectively. For the local discriminator, patches are randomly cropped from both the real images and the generated outputs. Unlike the global discriminator, the local discriminator utilizes the LSGAN loss, with the values calculated for each of the patches averaged and applied. The equations for the loss functions applied to the local discriminator *D* and the generator *G* are as follows:

(3) $$L_{D}^{Local}=E_{x_{r}\sim P_{real-patches}}\left[{\left(D\left(x_{r}\right)-1\right)}^{2}\right]+E_{x_{f}\sim P_{fake-patches}}[{\left(D(x_{f})\right)}^{2}]$$

(4) $$L_{G}^{Local}=E_{x_{r}\sim P_{fake-patches}}\left[{\left(D\left(x_{r}\right)-1\right)}^{2}\right]$$

In Equations (3) and (4), *D* represents the network of the local discriminator, while $x_{r}$ and $x_{f}$ denote randomly cropped sample patches from the real images and fake images, respectively. In summary, Equation (1) is used to train the global discriminator, Equation (3) is employed to train the local discriminator, and Equations (2) and (4) are used to train the generator.

### 3.3. Perceptual Loss

Johnson et al. [29] proposed perceptual loss for image transformation tasks to preserve style between images. Instead of relying on per-pixel differences, this method uses a pre-trained VGG-16 [30] model to calculate the distance between image features, thereby maintaining visual quality. Perceptual loss has been widely applied and proven effective in low-level vision tasks such as image super-resolution, low-light enhancement, and style transfer.

In this study, since unpaired data is used, the distribution of enhanced images during adversarial training may converge to that of unrelated images, independent of the input images. This limitation of unsupervised learning can cause the output images to appear contextually and semantically different from the originals. Perceptual loss constrains the distance between the features of the input and output images, playing a crucial role in ensuring that Squeeze U-net preserves the context of the original images. Notably, prior studies [10,31] have demonstrated the VGG model’s robustness to variations in image illumination, making it particularly suitable for this task. The corresponding equation is as follows:

(5) $$L_{perceptual}=\frac{1}{W_{i,\;j}H_{i,\;j}}\sum_{x=1}^{W_{i,\;j}}\sum_{y=1}^{H_{i,\;j}}{\left(\phi_{i,\;\;j}{\left(I^{L}\right)}_{x,y}-\phi_{i,\;\;j}{\left(G\left(I^{L}\right)\right)}_{x,y}\right)}^{2}$$

In Equation (5), *G* represents the generator, and $I^{L}$ denotes the low-light image input to the generator. ϕ refers to the feature map extracted from a pre-trained VGG-16 model, where *i* indicates the *i*-th max-pooling layer of VGG-16, and *j* represents the *j*-th convolutional layer following the *i*-th max-pooling layer. $W_{i,\;j}$ and $H_{i,\;j}$ denote the dimensions of the extracted feature map. By default, we set *i* = 1 and *j* = 5. Similarly, Equation (5) is also applied to cropped patches used as inputs for the local discriminator. Additionally, an instance normalization layer was added before feeding inputs into the VGG-16 model.

## 4. Experiments

### 4.1. Datasets and Two Training Sessions

We supplemented the datasets used in EnlightenGAN with additional datasets focused on road scenes to address driving scenarios. Specifically, we utilized the “High-Precision Data Collection Vehicle Night City Road Data” (HP-NCR) [32] and the “High-Precision Data Collection Vehicle Daytime City Road Data” (HP-DCR) [33]. Both datasets are publicly available on the AI-Hub website managed by the National Information Society Agency (NIA), a governmental organization in South Korea.

The HP-NCR and HP-DCR datasets consist of images extracted from videos captured at a resolution of 1920 × 1080 using cameras mounted on vehicles driving through urban roads at night and during the day, respectively. The images were extracted at 10 fps from 20-s videos, with 500 driving scenes resulting in over 100,000 images per dataset. However, this study utilized only a subset of these images. The driving scenes were recorded under various weather conditions (clear, cloudy, and rainy). Notably, the HP-NCR dataset exhibits extremely dark characteristics due to its low exposure settings.

The training dataset used for Squeeze-EnGAN is the same as that used in previous research on EnlightenGAN [34,35,36,37]. During the training process, 1013 low-light images were used as inputs for the generator, while 1102 normal-light images were used as reference targets. Additionally, we designed a new training session using a modified dataset. Thanks to the flexibility of unsupervised learning, various datasets can be effectively applied, leading to improved domain adaptation performance. This training session was conducted to verify whether the model could capture diverse real-world characteristics. Specifically, we aimed to learn domain-specific characteristics of road scenes by leveraging the HP-NCR and HP-DCR datasets. In this training session, the low-light input images for the generator consisted of 500 images from the low-light dataset used in EnlightenGAN and 1000 selected images from the HP-NCR dataset, resulting in a combined set of 1500 images. For normal-light images, 1102 images from the EnlightenGAN training dataset were combined with 398 images from the HP-DCR dataset. In this study, we name the model derived from this training session Squeeze-EnGAN-R. In all training sessions, images were resized to a resolution of 640 × 400.

**Figure 4:**
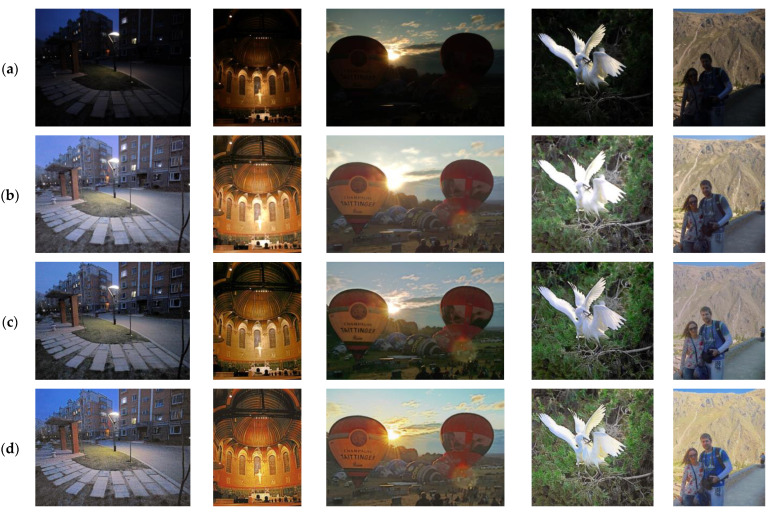

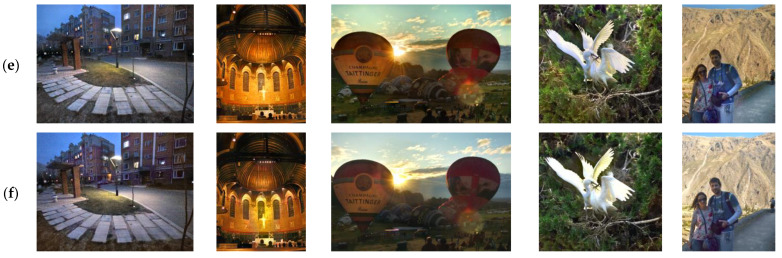
Visualization of comparisons between the original images and the outcomes of the models for the LIME, DICM, MEF, NPE, and VV image sets. (**a**) Input. (**b**) URetinex-Net. (**c**) ZeroDCE. (**d**) PairLIE. (**e**) EnlightenGAN. (**f**) Squeeze-EnGAN (Ours).

**Figure 5:**
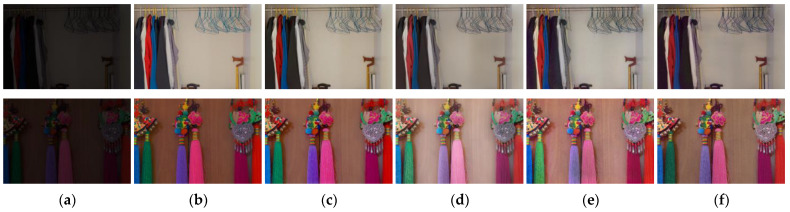
Visualization of comparisons between the original images and the outcomes of the models for the LOL dataset. (**a**) input. (**b**) Ground Truth. (**c**) CIDNet. (**d**) LLFlow. (**e**) EnlightenGAN. (**f**) Squeeze-EnGAN (Ours).

**Figure 6:**
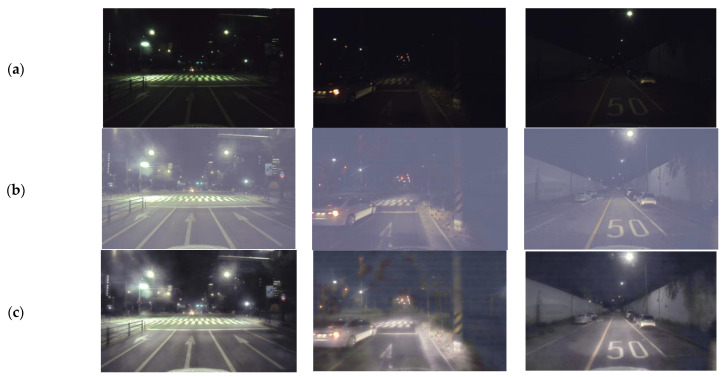

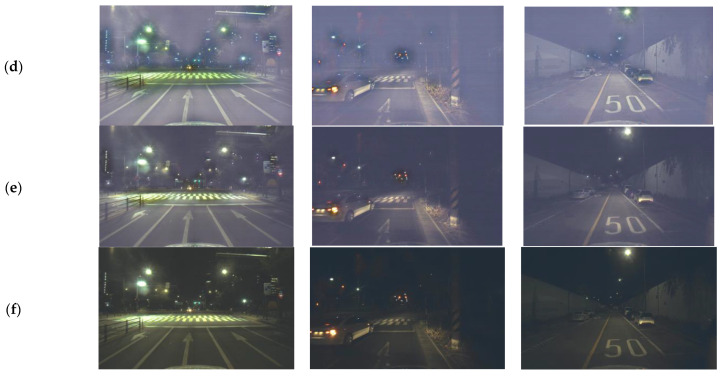
Visualization of comparisons between the original images and the outcomes of the models for the HP-NCR image sets. (**a**) Input. (**b**) URetinexNet. (**c**) LLFlow. (**d**) EnlightenGAN. (**e**) Squeeze-EnGAN. (**f**) Squeeze-EnGAN-R.

For testing, we employed datasets used in previous studies (DICM [38], MEF [39], NPE [40], LIME [41], VV [42], and LOL [34]) along with 554 selected images from the HP-NCR dataset. The model outputs for the MEF, NPE, LIME, and VV datasets are shown in Figure 4, while the results for the LOL dataset are presented in Figure 5, and those for the HP-NCR dataset are shown in Figure 6.

### 4.2. Implementation Details

Our model was implemented using the PyTorch 1.12.1 library and trained in an environment configured with CUDA 11.3 and Python 3.9.17. The training of Squeeze-EnGAN was conducted over a total of 200 epochs. Initially, the model was trained for 100 epochs with a learning rate of 1 × 10^−4^, followed by a linearly decayed learning rate for the remaining 100 epochs. The local discriminator was configured to use 6 patches, each with a size of 64 × 64. In this study, we set the batch size to 24 and utilized the Adam Optimizer [43] for training. The entire training process was performed using a single NVIDIA A6000 GPU, requiring a total of 4 h 30 min to complete. In the case of Squeeze-EnGAN-R, the larger dataset increased the training time to 6 h 40 min.

### 4.3. Results

We performed comparative analyses with previous methods. Among them, URetinex-Net [44], CIDNet [45], and LLFlow [46] are supervised learning models, while ZeroDCE [47], PairLIE [48], and EnlightenGAN are unsupervised learning models. In Figure 4, which compares the result images of different models, the first to last columns represent the results for the LIME, DICM, MEF, NPE, and VV datasets, respectively. As shown in Figure 4f, replacing convolution layers with lightweight fire modules still yields high-quality results compared to other models. To compare the output quality of Squeeze-EnGAN with that of existing studies, we evaluated the average NIQE (Natural Image Quality Evaluator) score [49] for each test dataset. NIQE is a well-known no-reference image quality assessment metric, enabling quality evaluation without the need for ground-truth images. It measures how closely the test image resembles the statistical characteristics of natural images, with lower scores indicating better quality. Table 2 presents the NIQE scores measured for the datasets used in previous studies (DICM, MEF, NPE, LIME, and VV) as well as the HP-NCR dataset.

According to Table 2, the enhanced images produced by Squeeze-EnGAN demonstrated higher quality compared to the original images across all image sets. While EnlightenGAN achieved high quality in terms of the NIQE metric due to its large number of parameters, Squeeze-EnGAN maintained a comparable performance despite being lightweight. The NIQE score difference between the two models was 0.08 for the VV test set with the smallest gap, while it reached 0.24 for the DICM test set with the largest difference. These results demonstrate that Squeeze-EnGAN achieves competitive performance in enhancing image quality under various test conditions.

Additionally, the PSNR (Peak Signal-to-noise ratio) and SSIM (Structural Similarity Index Measure) [50] for the LOL dataset were evaluated and summarized in Table 3. A higher PSNR value indicates better reconstruction quality with lower distortion. Meanwhile, SSIM ranges from 0 to 1, with values closer to 1 indicating higher structural similarity to the reference image. Although our model achieves lower scores than supervised learning models in both metrics, Figure 5 shows that it produces visually high-quality results. Furthermore, our model achieved a higher SSIM score than the EnlightenGAN, even with fewer parameters.

For the HP-NCR dataset, we compared the results using the Squeeze-EnGAN-R model, which was trained to capture the characteristics of road scenes. As a result, the Squeeze-EnGAN-R model produced images with less noise and blur than both the original and other models. As shown in Figure 6, EnlightenGAN introduces artifacts and generates unnatural images for driving scenes. Other models also lead to unnatural color distortions and excessive blurring caused by over-enhancement of brightness. On the other hand, the results of Squeeze-EnGAN-R exhibit more natural colors, enabling the detection model to identify cars more effectively.

High-level vision tasks, such as identifying surrounding objects, play a critical role in camera-based perception modules for autonomous vehicles and intelligent mobility systems. To verify whether our low-light enhancement process contributes to object identification tasks, we conducted an effectiveness evaluation. Specifically, we compared the detection performance of the original images from the HP-NCR dataset with the enhanced outcomes. The 554 test images from the HP-NCR dataset contained a total of 823 vehicles, and we detected these vehicles using YOLO11 [51], a well-known object detection model. From the YOLO11 models available in different parameter sizes, we selected and utilized the small (s) and medium (m) models. The detection performance was evaluated using Precision, Recall, and mAP, with the results presented in the accompanying Table 4. These findings demonstrate the impact of low-light enhancement on improving object detection in challenging nighttime conditions.

According to Table 4, the detection performance improved across all metrics in the results of both EnlightenGAN and Squeeze-EnGAN-R compared to the original images. Notably, the results of Squeeze-EnGAN-R demonstrated better detection performance than those of EnlightenGAN across most metrics. In particular, the outcomes of Squeeze-EnGAN-R showed better performance across all metrics when using the YOLO11-s model. Even when compared to the original Squeeze-EnGAN model, Squeeze-EnGAN-R achieved higher performance in most metrics. This indicates that Squeeze-EnGAN-R adapts more effectively to driving environments and suggests that using diverse datasets in Squeeze-EnGAN can improve its performance in specific scenarios. In particular, unsupervised learning models are well suited for applications such as continual learning frameworks [52], where model weights are dynamically updated in real time to adapt to diverse driving environments.

### 4.4. Performance on Edge System

Most mobility systems face constraints in power supply and space, making it impractical to use computers equipped with high-performance GPUs. Additionally, installing expensive computing equipment in vehicles poses challenges in mass production due to cost concerns. With the recent advancements in compact onboard computers, GPU-equipped single-board computers are increasingly used in research for autonomous driving and robotics control to handle AI computations.

To demonstrate the utility of our model in mobility edge system environments, we measured inference times on NVIDIA’s GPU-equipped single-board computers, namely Jetson Xavier AGX (32 GB Developer Kit) and Jetson Nano (2 GB Developer Kit). The models were executed in a PyTorch 1.10 environment supported by the JetPack SDK. As shown in Table 5, Squeeze-EnGAN achieved faster inference speed than LLFlow, CIDNet, URetinex-Net, PairLIE, and EnlightenGAN on both devices. Additionally, due to memory limitations, LLFlow could not perform stable inference on the Jetson Nano. Notably, the Squeeze-EnGAN model achieves a 30.68% reduction in total inference time on the Jetson Xavier compared to EnlightenGAN. On the other hand, Squeeze-EnGAN demonstrated a 10% faster inference time on the Jetson Nano. During the model execution, the GPU memory usage measured on the Jetson Nano was 721 MB for Squeeze-EnGAN, while EnlightenGAN consumed 934 MB, which is 29.55% higher. In addition to these evaluations, the lower MACs observed in Table 1 further indicate a reduction in the model’s computational complexity. This provides insights into the model’s feasibility for deployment in resource-constrained, real-world mobility applications.

### 4.5. Ablation Study

We conducted an ablation study to investigate the effects of each component of Squeeze-EnGAN by performing new training sessions with certain components removed or modified. Specifically, experiments were conducted under the following four conditions:

1. To investigate the effect of the local discriminator, we conducted training without it.
2. All layers of the generator network shown in Figure 2, except for the final convolution layer, were replaced with fire modules.
3. Fire modules in the decoder were removed and replaced with standard convolution layers. Therefore, fire modules are used only in the encoder.
4. Fire modules in the encoder were removed and replaced with standard convolution layers. Therefore, fire modules are used only in the decoder.

**Figure 7:**
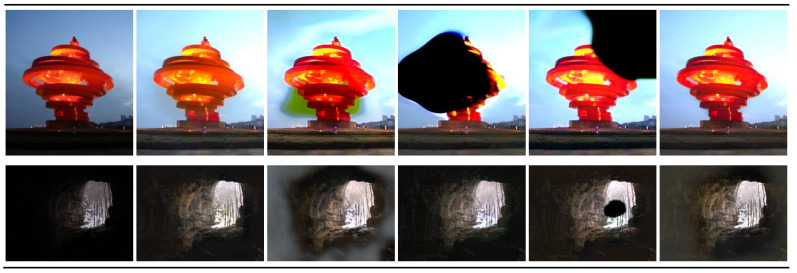

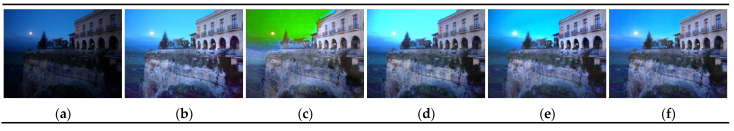
Comparison of result images for the original, Squeeze-EnGAN, and ablation experiments. Each column represents the results for the following: (**a**) input, (**b**) Squeeze-EnGAN, (**c**) training without the local discriminator, (**d**) replacing all generator layers with fire modules except the final convolution layer, (**e**) using fire modules only in the encoder, and (**f**) using fire modules only in the decoder.

The training dataset was not modified, and the experiments were conducted under the same conditions. The result images from each experiment are presented in Figure 7.

The results in Figure 7c demonstrate that excluding the local discriminator leads to a significant degradation in the quality of the output images. Although the brightness of the images has been enhanced, blurring and artifacts can be observed in all three images. In the case of Figure 7d, the image in the first row shows large artifacts on the structure. This indicates that using the fire module for the input image fails to extract sufficient features. Similarly, blurring and artifacts can be observed in the images of Figure 7e. This suggests that despite the high representational capacity of the decoder, significant information loss occurs during the encoding process. This results in suboptimal reconstruction, likely due to insufficient preservation of fine-grained spatial details in the encoder.

In contrast, Figure 7f exhibit realistic colors and visually high-quality images. This suggests that the rich information extracted by the encoder can be effectively processed by the fire module. However, as shown in Table 1, it requires approximately 3.9 M additional parameters compared to Squeeze-EnGAN. This indicates that the total number of parameters is more than approximately 3.8 times that of Squeeze-EnGAN. Overall, as shown in Figure 7b, Squeeze-EnGAN can generate high-quality images with a limited number of parameters. This supports the idea that our model achieves high performance while remaining lightweight by appropriately utilizing the local discriminator and strategically placing the fire modules.

## 5. Conclusions

This paper addresses methods to overcome the performance degradation of RGB cameras in low-light environments and enhance the perceptual capabilities of intelligent mobility systems. We focused on an unsupervised learning-based low-light image enhancement method using unpaired data. Furthermore, we proposed a lightweight deep learning model considering real-time applicability in driving environments. The model is designed as a GAN-based generator-discriminator network, enabling training without paired image data. To achieve model optimization, we incorporated fire modules into the U-net structure of the generator, significantly reducing the number of parameters and computational complexity.

Despite the lightweight design, the enhanced images produced by the model demonstrated visually superior quality compared to the original images and achieved competitive scores on the NIQE metric. Furthermore, our experiments confirmed an improvement in object detection rates in real driving scenes, demonstrating the model’s effectiveness for intelligent mobility applications. Notably, Squeeze-EnGAN achieved an inference time of 0.061 s on a compact onboard PC, delivering real-time performance while maintaining results comparable to those of existing studies. These findings suggest that the proposed method is well-suited for mobility environments requiring rapid decision-making to avoid accidents and provides a critical solution for enhancing the safety of future driving technologies. Nevertheless, to further optimize the system for autonomous driving applications, the overall framework must be refined. For instance, research should be conducted on middleware systems to ensure stable frame acquisition from cameras, process scheduling, and hardware accelerators to enhance computational efficiency.

Squeeze-EnGAN leverages an unsupervised learning approach, offering the significant advantage of training with unpaired image sets. However, the use of a GAN-based architecture presents challenges in balancing the generator and discriminator. Additionally, the model’s performance is highly dependent on the quality of the training dataset. In this study, we utilized the HP-NCR dataset, which contains various nighttime driving scenes and is publicly available through AI-Hub. However, noise and aliasing were observed in the source images, potentially impacting performance. To further enhance the model’s capabilities, it will be necessary to curate high-quality training datasets that effectively represent diverse driving scenarios or apply appropriate preprocessing steps to the data prior to model input. These improvements could significantly bolster the robustness and reliability of the proposed method.

Future research should focus on identifying optimal datasets while also exploring methods to further accelerate inference speed. Since intelligent driving systems are responsible for transporting people and goods, they must completely mitigate the risk of accidents. Enhancing low-light images should not become a double-edged sword that wastes resources and overloads the system. Achieving robust performance across diverse environmental conditions while minimizing resource consumption will significantly enhance the reliability of future driving technologies. These advancements are critical for ensuring both the efficiency and safety of intelligent mobility systems.

## Figures and Tables

**Table 1:** Comparison of #MACs and the number of parameters between the generator with the fire module (ours) and the generator of EnlightenGAN. The input image size for each network is the same at 640 × 400.

Layer Name	Squeeze-EnGAN (Ours)			EnlightenGAN *		
	Components	#MACs (M)	#Params	Components	#MACs (M)	#Params
Conv1	Conv2D × 2	2646.016	10272	Conv2D × 2	2719.744	10560
DS1	Maxpooling × 1 Fire module × 2	1556.480	24128	Maxpooling × 1 Conv2D × 2	3571.712	55680
DS2	Maxpooling × 1 Fire module × 2	1153.024	71712	Maxpooling × 1 Conv2D × 2	3555.328	221952
DS3	Maxpooling × 1 Fire module × 2	763.904	190336	Maxpooling × 1 Conv2D × 2	3547.136	886272
DS4	Maxpooling × 1 Fire module × 2	475.776	474592	Maxpooling × 1 Conv2D × 2	3543.040	3542016
US1	Upsampling × 1 Fire module × 3	1459.520	358608	Upsampling × 1 Conv2D × 3	11828.224	2950912
US2	Upsampling × 1 Fire module × 3	2266.112	138464	Upsampling × 1 Conv2D × 3	11859.968	738176
US3	Upsampling × 1 Fire module × 3	3226.624	48816	Upsampling × 1 Conv2D × 3	11923.456	184768
US4	Upsampling × 1 Conv2D × 3	12034.048	46240	Upsampling × 1 Conv2D × 3	12034.048	46240
Conv2	Conv2D × 1	25.344	99	Conv2D × 1	25.344	99
Total		25606.848	1363267		64608.000	8636675

* The generator in EnlightenGAN incorporates an attention module.

**Table 2:** Comparison of NIQE metrics for different models on the DICM, MEF, NPE, LIME, VV, and HP-NCR dataset.

Method	Dataset					
	DICM	MEF	NPE	LIME	VV	HP-NCR
Input	4.25	4.27	4.32	4.35	3.52	7.02
URetinex-Net	4.20	3.79	4.69	4.34	3.03	6.34
CIDNet	3.79	3.56	3.74	4.13	3.21	5.82
LLFlow	3.63	3.46	4.09	3.98	3.01	5.69
ZeroDCE	4.58	4.93	4.53	5.82	4.81	5.95
PairLIE	4.09	4.18	4.21	4.51	3.66	5.91
EnlightenGAN	3.50	3.23	4.11	3.72	2.58	5.48
Ours	3.74	3.37	4.29	3.89	2.66	5.68

**Table 3:** Comparison of PSNR and SSIM metrics for different models for the LOL dataset. SL means supervised learning, while UL means unsupervised learning.

Method		LOL Dataset	
		PSNR	SSIM
SL	URetinex-Net	21.328	0.835
	CIDNet	23.809	0.857
	LLFlow	21.149	0.854
UL	PairLIE	19.510	0.736
	ZeroDCE	14.861	0.559
	EnlightenGAN	17.480	0.651
	Ours	16.174	0.658

**Table 4:** Vehicle detection performance on the HP-NCR dataset and enhanced outcomes measured using YOLO11.

Metric		Method			
		Original	EnlightenGAN	Squeeze-EnGAN	Squeeze-EnGAN-R
YOLO11-s	Precision	0.364	0.536	0.377	0.537
	Recall	0.369	0.329	0.436	0.375
	mAP50	0.335	0.372	0.387	0.399
	mAP50-95	0.183	0.192	0.195	0.208
YOLO11-m	Precision	0.622	0.645	0.710	0.623
	Recall	0.394	0.418	0.410	0.453
	mAP50	0.450	0.485	0.490	0.497
	mAP50-95	0.264	0.282	0.288	0.290

**Table 5:** Comparison of inference times for each model on two devices with a 600 × 400 image input.

Method	Inference Time (s)	
	Jetson Xavier	Jetson Nano
LLFlow	1.612	-
CIDNet	0.241	1.760
URetinex-Net	0.371	3.332
PairLIE	0.157	1.350
EnlightenGAN	0.088	0.651
Squeeze-EnGAN (ours)	0.061	0.590

## Data Availability

The data are available upon request to the authors.

## Appendix A

This section describes the detailed architecture of the generator. Table A1 shows the layer specifications for the encoder part of the generator, while Table A2 contains the layer specifications for the decoder part of the generator. After every convolution operation, including those within the fire modules, LeakyReLU and batch normalization layers are applied. However, batch normalization is not applied to the single convolution operation or single fire module that follows an upsampling step. The final output is combined with the input and clamped to the range of −1.0 to 1.0.

**Table A1:** The layer specifications for the encoder part of the generator.

Layer	C_in_	C_mid_	C_out_	Kernel Size
Convolution 2D	3	-	32	3 × 3
Convolution 2D	32	-	32	3 × 3
MaxPooling	-	-	-	2 × 2
FireModule	32	32	64	3 × 3
FireModule	64	32	64	3 × 3
MaxPooling	-	-	-	2 × 2
FireModule	64	48	128	3 × 3
FireModule	128	48	128	3 × 3
MaxPooling	-	-	-	2 × 2
FireModule	128	64	256	3 × 3
FireModule	256	64	256	3×3
MaxPooling	-	-	-	2×2
FireModule	256	80	512	3×3
FireModule	512	80	512	3×3

**Table A2:** The layer specifications for the decoder part of the generator.

Layer	C_in_	C_mid_	C_out_	Kernel Size
Upsampling	-	-	-	-
FireModule	512	80	256	3 × 3
FireModule	512	64	256	3 × 3
FireModule	256	64	256	3 × 3
Upsampling	-	-	-	-
FireModule	256	64	128	3 × 3
FireModule	256	48	128	3 × 3
FireModule	128	48	128	3 × 3
Upsampling	-	-	-	-
FireModule	128	48	64	3 × 3
FireModule	128	32	64	3 × 3
FireModule	64	32	64	3 × 3
Upsampling	-	-	-	-
Convolution 2D	64	-	32	3 × 3
Convolution 2D	64	-	32	3 × 3
Convolution 2D	32	-	3	3 × 3
